# Author Correction: Regulatory analysis of single cell multiome gene expression and chromatin accessibility data with scREG

**DOI:** 10.1186/s13059-022-02786-9

**Published:** 2022-10-13

**Authors:** Zhana Duren, Fengge Chang, Fnu Naqing, Jingxue Xin, Qiao Liu, Wing Hung Wong

**Affiliations:** 1 Center for Human Genetics and Department of Genetics and Biochemistry, Clemson University, Greenwood, SC 29646 USA; 2 Department of Statistics, Department of Biomedical Data Science and Bio-X Program, Stanford University, Stanford, CA 94305 USA

**Correction: Genome Biol 23, 114 (2022)**

**https://doi.org/10.1186/s13059-022-02682-2**

Following publication of the original paper [1], the authors have reported an error in reference genome version of the HiC data used for validation of the RE-TG interactions. After using the correct version of the HiC anchor locations, the result in Fig. 4D, supplementary Figure S11, and S12 are all improved.

**Fig. 4:**
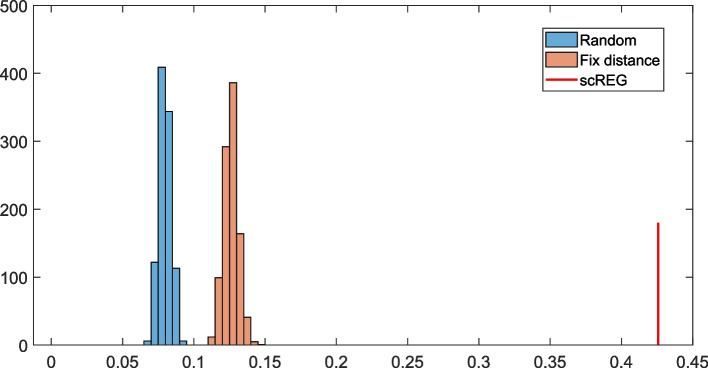
**D**. Validation of RE-TG prediction by HiC data on Naïve CD4 T cell

## Supplementary Information

**Additional file 1: Supplementary Figure S11**. Validation of RE-TG prediction by HiC data. Consistency ratio of predicted RE and promoter capture HiC data on different cell types of. We can see in all cell type, scREG predict the greatest number of same RE-TG pairs as previously found promoter capture HiC data. set select distribution distance same with scREG, does improve the performance. **Supplementary Figure S12**. AUROC and AUPR of RE-TG predictio by taking HiC data as ground truth.
