# Supplementary material for: Regulatory analysis of single cell multiome gene expression and chromatin accessibility data with scREG
Source: Genome Biol. 2022 May 16;23:114. doi: 10.1186/s13059-022-02682-2 (PMC9109353; doi:10.1186/s13059-022-02682-2)
Supplement: Supplementary file 1 — Additional file 1: Figure S1. Comparison of SI resulting from different methods. Figure S2. UMAP visualization of scAI and MOFA+ clustering. Figure S3. Performance of different Methods under different resolutions. Figure S4. UMAP visualization of marker genes’ expression on mouse E18 brain data. Figure S5. UMAP visualization of marker genes’ expression on human cerebellum data. Figure S6. UMAP visualization of marker genes’ expression on lymph node from B cell lymphoma data. Figure S7. Evaluation of Seurat V4 clustering and scREG clustering on Cell Ranger scRNA-seq embedding and Cell Ranger scATAC-seq embedding on mouse E18 brain. Figure S8. Evaluation of Seurat V4 clustering and scREG clustering on Cell Ranger scRNA-seq embedding and Cell Ranger scATAC-seq embedding on human cerebellum. Figure S9. Evaluation of Seurat V4 clustering and scREG clustering on Cell Ranger scRNA-seq embedding and Cell Ranger scATAC-seq embedding on lymph node from B cell lymphoma. Figure S10. UMAP visualization of BMMC cell using Seurat and scREG clustering. Figure S11. Validate the RE-TG prediction by HiC data. Figure S12. AUROC and AUPR predicting HiC data. Figure S13. Clusters specificity of CRS. [file 13059_2022_2682_MOESM1_ESM.pdf]

Additional file 1

S1

A

	RNA_PCA	ATAC_LSA	PCA+LSA	scAI	MOFA+	intNMF	scREG
CD56 (bright) NK cells	0.3987	0.6764	0.2341	0.7878	0.6634	0.8503	0.8868
CD56 (dim) NK cells	0.1569	0.5368	0.0441	0.5719	0.5249	0.7374	0.6856
MAIT T cells	0.3640	0.3738	0.4244	0.4226	0.4191	0.5627	0.5860
classical monocytes	0.3169	0.2292	0.3394	0.4271	0.2749	0.4282	0.3902
effector CD8 T cells	-0.2735	-0.1500	-0.2923	-0.3481	-0.2563	-0.5122	-0.4786
intermediate monocytes	0.2591	0.1585	0.2183	0.1886	0.2075	0.2172	0.3442
memory B cells	-0.0306	0.1653	0.0009	0.5803	0.4192	0.5237	0.4722
memory CD4 T cells	-0.0253	0.2397	-0.1283	0.1611	0.1938	0.4093	0.2871
myeloid DC	0.2107	0.3413	0.1495	0.5342	0.5855	0.4194	0.4405
naive B cells	0.5683	0.4573	0.5404	0.8610	0.6080	0.9109	0.9283
naive CD4 T cells	0.1766	0.4271	0.1337	0.3520	0.4161	0.3612	0.7410
naive CD8 T cells	0.0090	0.3799	-0.0265	0.2290	0.4395	0.4778	0.8081
non-classical monocytes	0.6352	0.5221	0.6259	0.8161	0.6831	0.8358	0.7920
plasmacytoid DC	0.7736	0.8785	0.6739	0.9885	0.9587	0.9328	0.9758
Average	0.2528	0.3740	0.2098	0.4694	0.4384	0.5110	0.5614

B

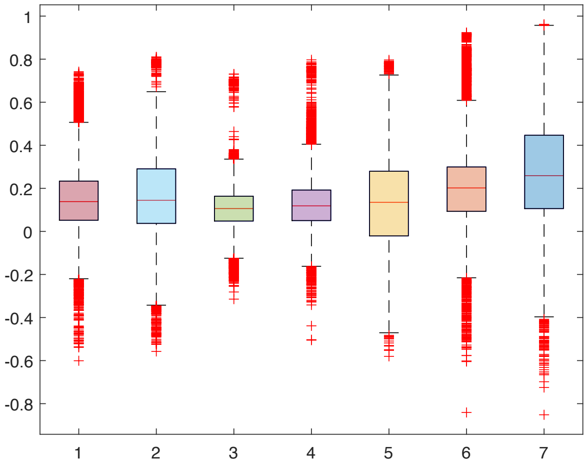

1. RNA_PCA,
2. ATAC_LSA,
3. RNA_PCA_and_ATAC_LSA(14D),
4. scAI,
5. MOFA+,
6. intNMF,
7. scREG

Fig S1. Comparison of SI resulting from different methods. (A) Colour scaled table compares the average Silhouette Index of each cell type when implementing different dimension reduction methods. We can see, all methods have poor performance on effector CD8 cell. Except for that, scREG performs better on most of the cell types, SI range from 0.2371to 0.9758. (B) Box plot shows the distribution of the average SI of all cells by different methods.

S2

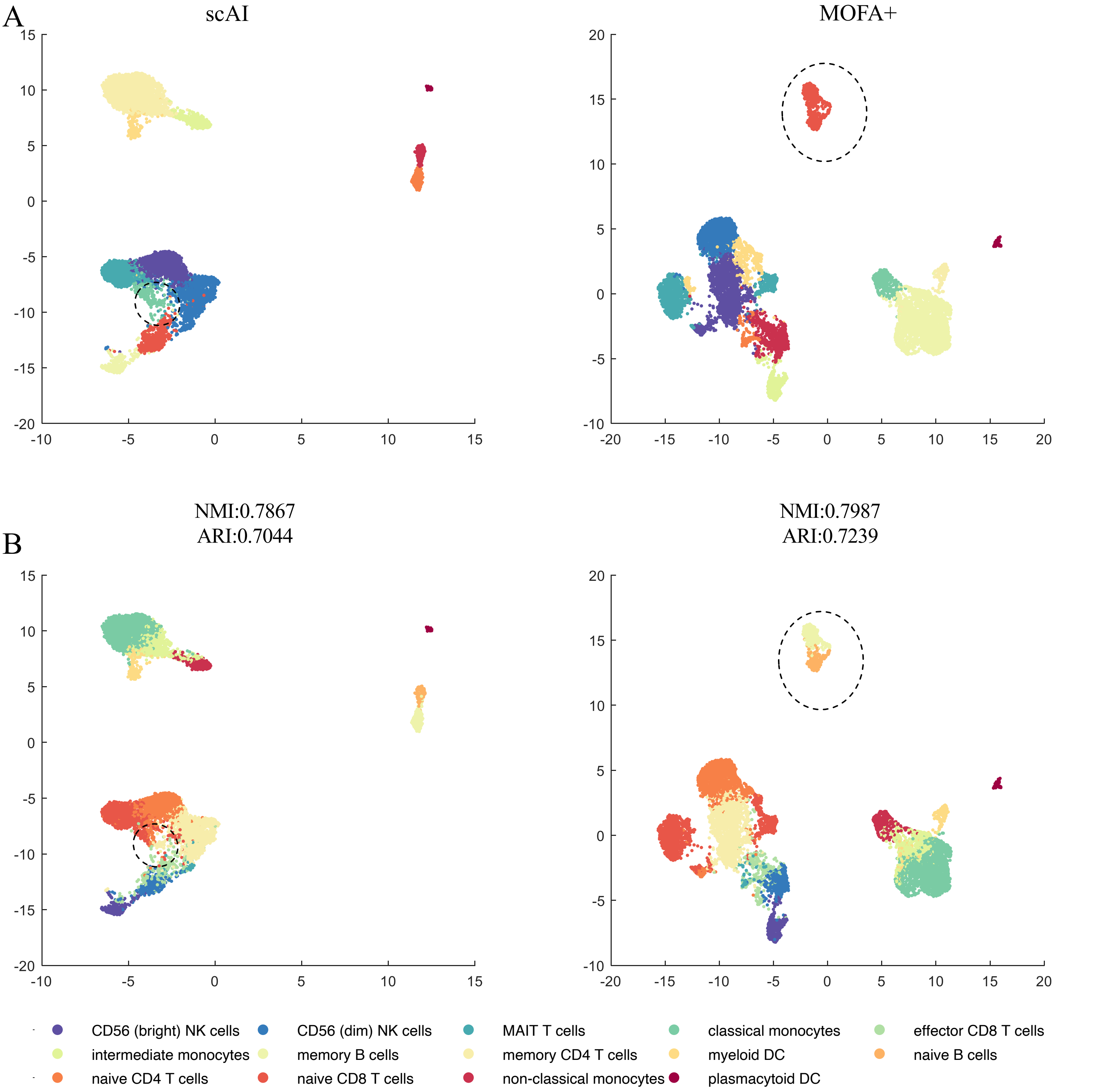

Fig S2. UMAP visualization of scAI and MOFA+ clustering. (A) Scatter plot visualize the Umap embedding colored by clustering label from different methods. (B) Same Umap as shown in (A) but colored by the surrogate ground truth. In scAI clustering, we see the cluster in circle is a mixture of three different clusters in true label. In MOFA+ clustering, memory B cells are not separated from naïve B cells; while this separation is clear in scREG. Clustering performance also assessed by calculating Normalized Mutual Information (NMI) and Adjusted Rand Index (ARI) based on the surrogate ground truth.

S3

Methods	intNMF		MOFA		scAI		Seurat		scREG	
	nmi	Ari	nmi	Ari	nmi	Ari	nmi	Ari	nmi	Ari
Resolution=0.2	0.7023	0.4158	0.7597	0.5466	0.7321	0.4467	0.8350	0.7694	0.7766	0.5886
Resolution=0.4	0.7901	0.6142	0.8039	0.7171	0.7627	0.5811	0.8303	0.7662	0.8337	0.7483
Resolution=0.6	0.8097	0.7141	0.7982	0.7114	0.7615	0.5803	0.7753	0.6351	0.8436	0.7765
Resolution=0.8	0.8035	0.7050	0.7977	0.7168	0.7845	0.6993	0.7607	0.6229	0.8417	0.7754
Resolution=1.0	0.7928	0.7056	0.7987	0.7239	0.7867	0.7044	0.7565	0.6206	0.8467	0.7881
Resolution=1.2	0.7868	0.6925	0.7634	0.6453	0.7879	0.7071	0.7551	0.6178	0.8286	0.7413
Resolution=1.4	0.7878	0.6944	0.7430	0.5377	0.7882	0.7076	0.7395	0.5613	0.8261	0.7384
Resolution=1.6	0.7903	0.6901	0.7477	0.5394	0.7860	0.7299	0.7410	0.5645	0.8186	0.7341
Resolution=1.8	0.7655	0.6435	0.7446	0.5332	0.7875	0.7325	0.7378	0.5658	0.8247	0.7345
Resolution=2.0	0.7638	0.6355	0.7451	0.5360	0.7891	0.7361	0.7360	0.5563	0.7932	0.6761

Fig S3. Performance of different Methods under different resolutions.
The clustering accuracy of all methods are sensitive to resolution change. The scREG performs very robust under different resolution parameters and achieve the highest performance among all the method we compared.

S4

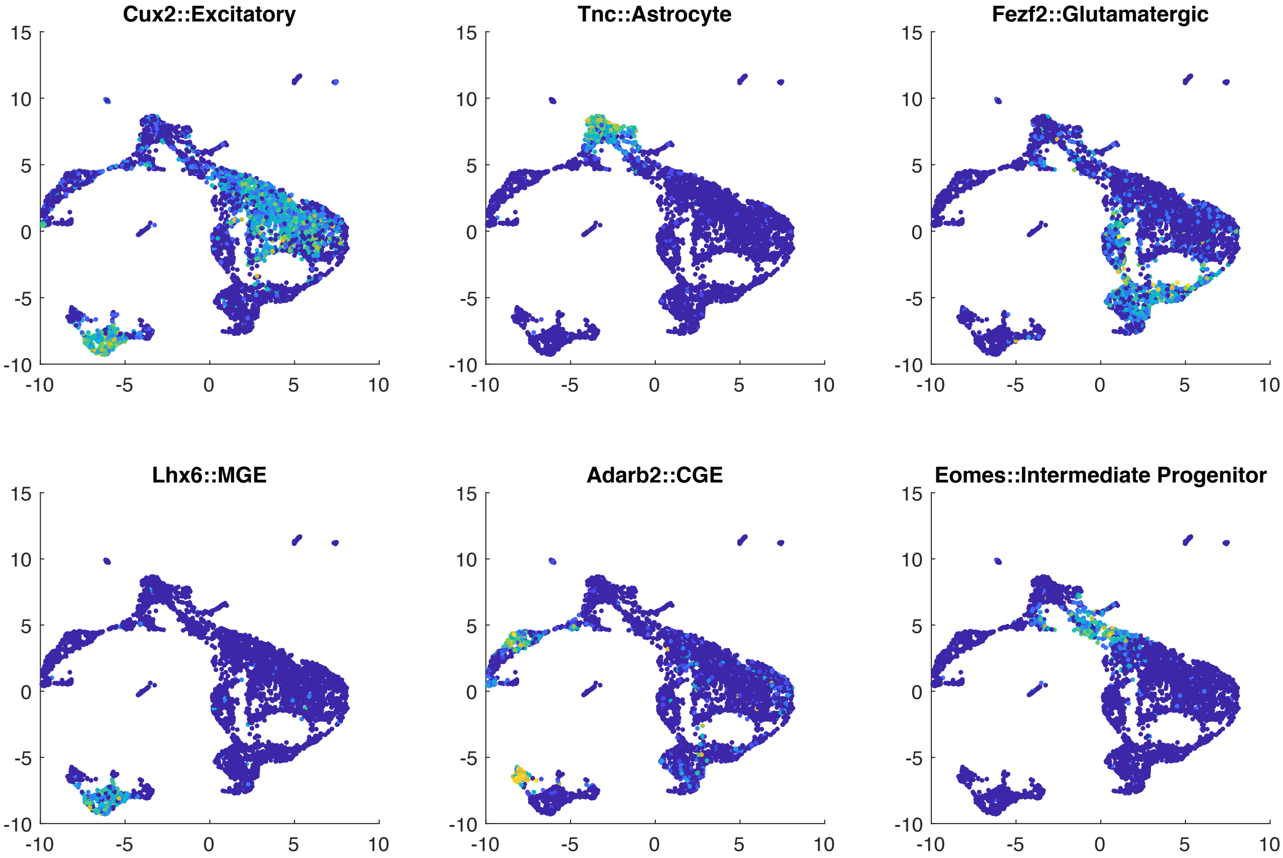

Fig S4. UMAP visualization of marker genes' expression on mouse E18 brain data. Each subfigure represents one marker genes' expression. The marker gene's name and corresponding cell type is written in top of each subfigure. Color represents the gene expression level on each cell. Clustering label is shown in Figure 3C.

S5

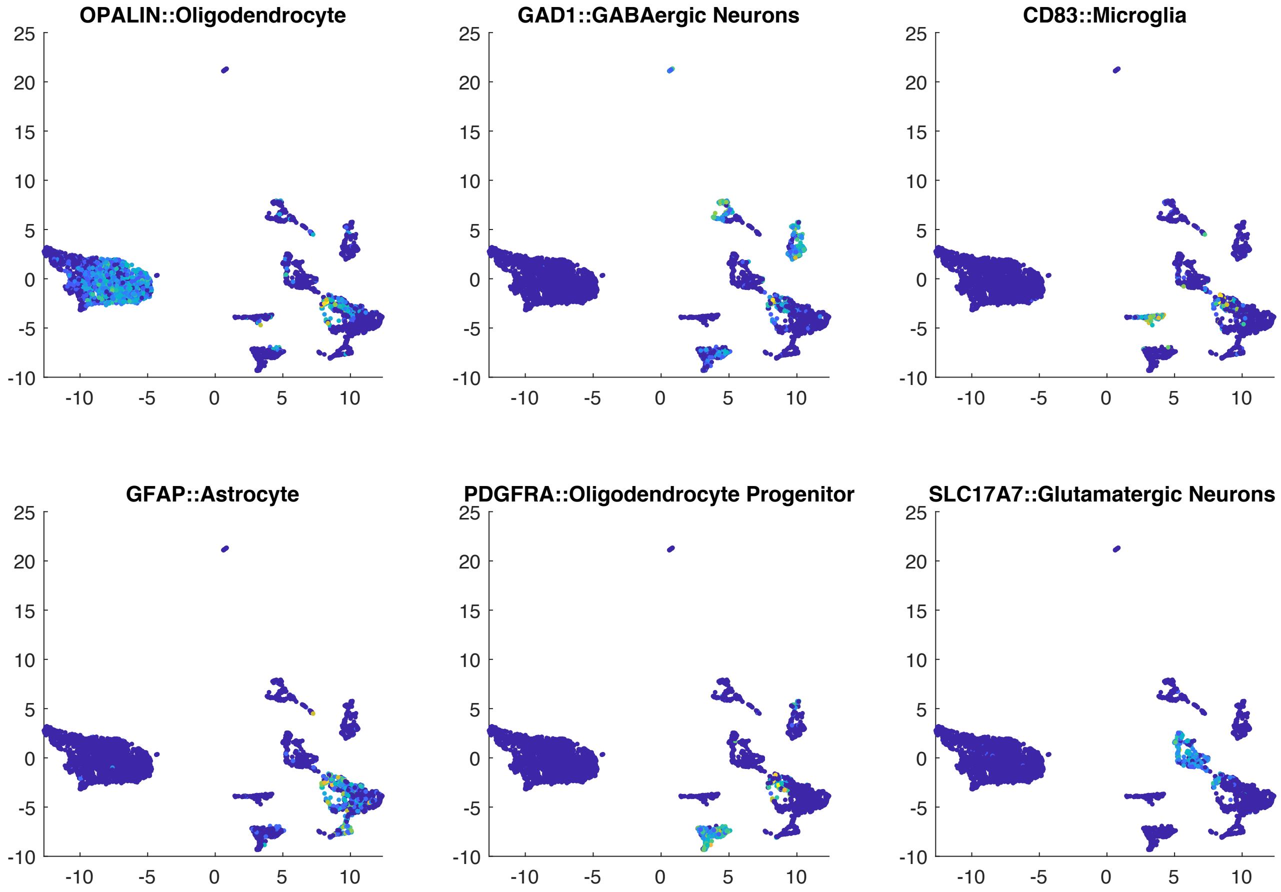

Figure S5. UMAP visualization of marker genes' expression on human cerebellum data. Each subfigure represents one marker genes' expression. The marker gene's name and corresponding cell type is written in top of each subfigure. Color represents the gene expression level on each cell. Clustering label is shown in Figure 3D.

S6

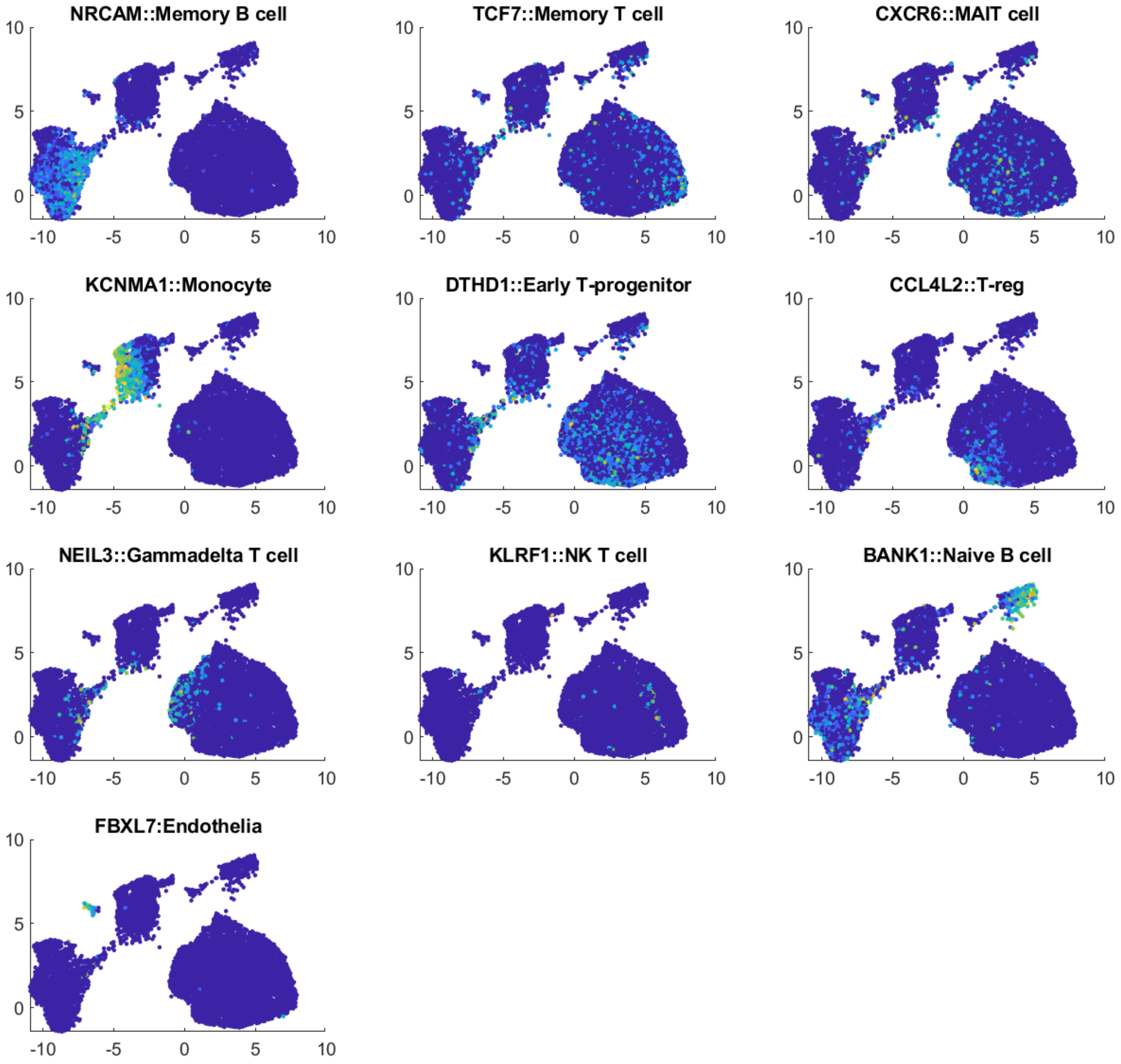

Figure S6. UMAP visualization of marker genes' expression on lymph node from B cell lymphoma data. Each subfigure represents one marker genes' expression. The marker gene's name and corresponding cell type is written in top of each subfigure. Color represents the gene expression level on each cell. Clustering label is shown in Figure 3E.

S7

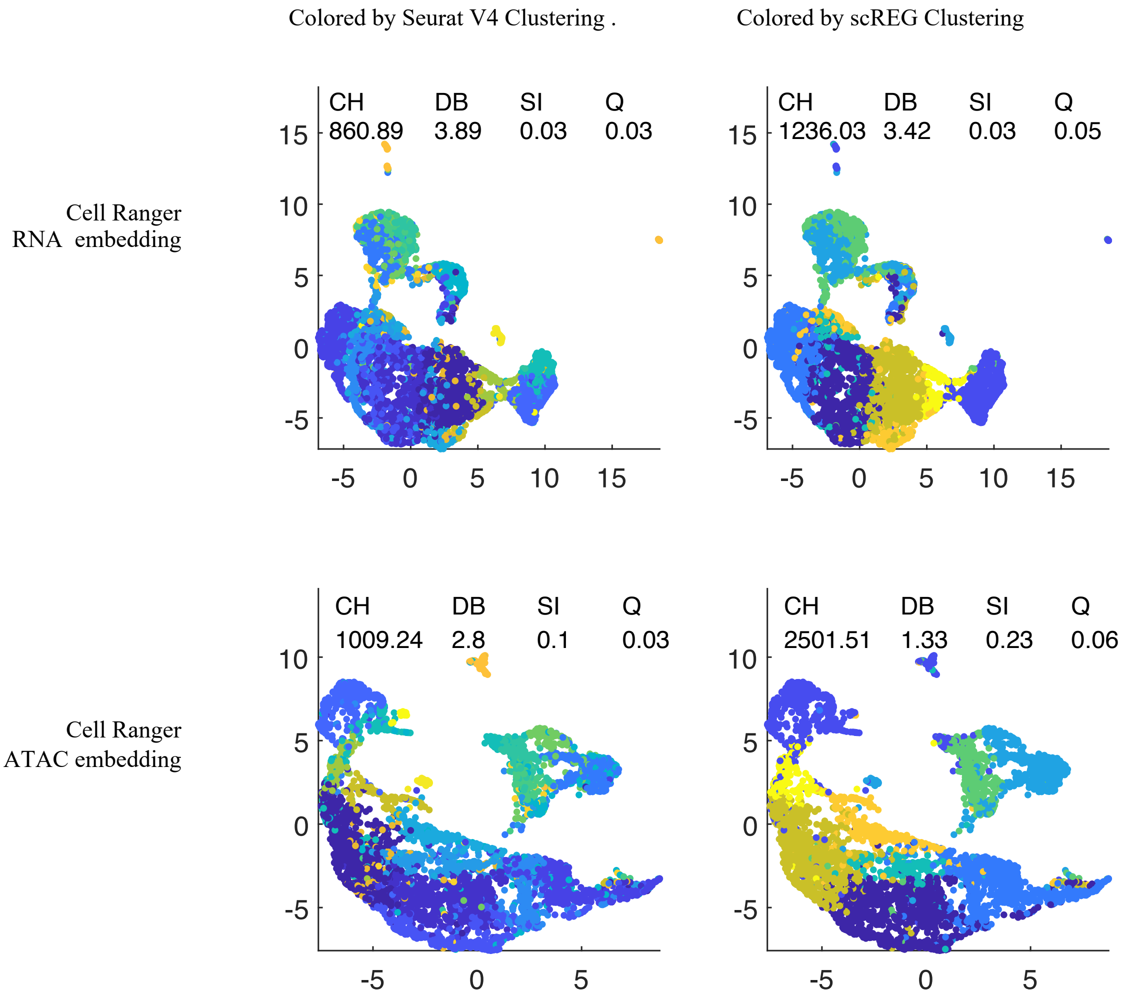

Figure S7. Evaluation of Seurat V4 clustering and scREG clustering on Cell Ranger scRNA-seq embedding and Cell Ranger scATAC-seq embedding on mouse E18 brain. Each row presents one common UMAP embedding, and each column represents one clustering method. The color of the dot represent the clustering label. In each subfigures, consistency between different clustering label and 20-dimension PCs embedding are measured by calculating Calinski-Harabasz Index (CH), Davies-Bouldin Index (DB), Silhouette Index (SI), Modularity Q value (Q) respectively. A lower Davies-Bouldin index indicate better clustering, but the other three metrics are the higher the better.

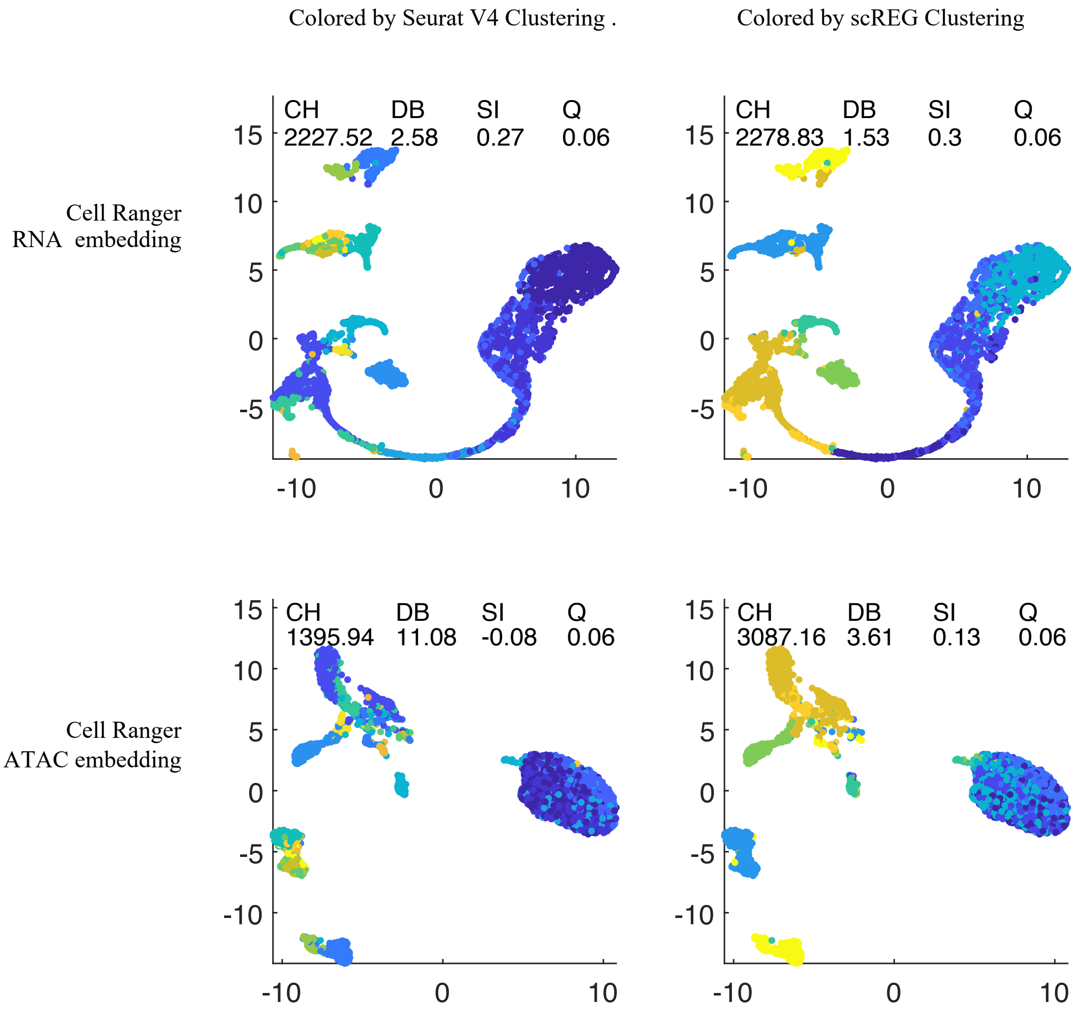

Figure S8. Evaluation of Seurat V4 clustering and scREG clustering on Cell Ranger scRNA-seq embedding and Cell Ranger scATAC-seq embedding on human cerebellum. Each row presents one common UMAP embedding, and each column represents one clustering method. The color of the dot represent the clustering label. In each subfigures, consistency between different clustering label and 20-dimension PCs embedding are measured by calculating Calinski-Harabasz Index (CH), Davies-Bouldin Index (DB), Silhouette Index (SI), Modularity Q value (Q) respectively. A lower Davies-Bouldin index indicate better clustering, but the other three metrics are the higher the better.

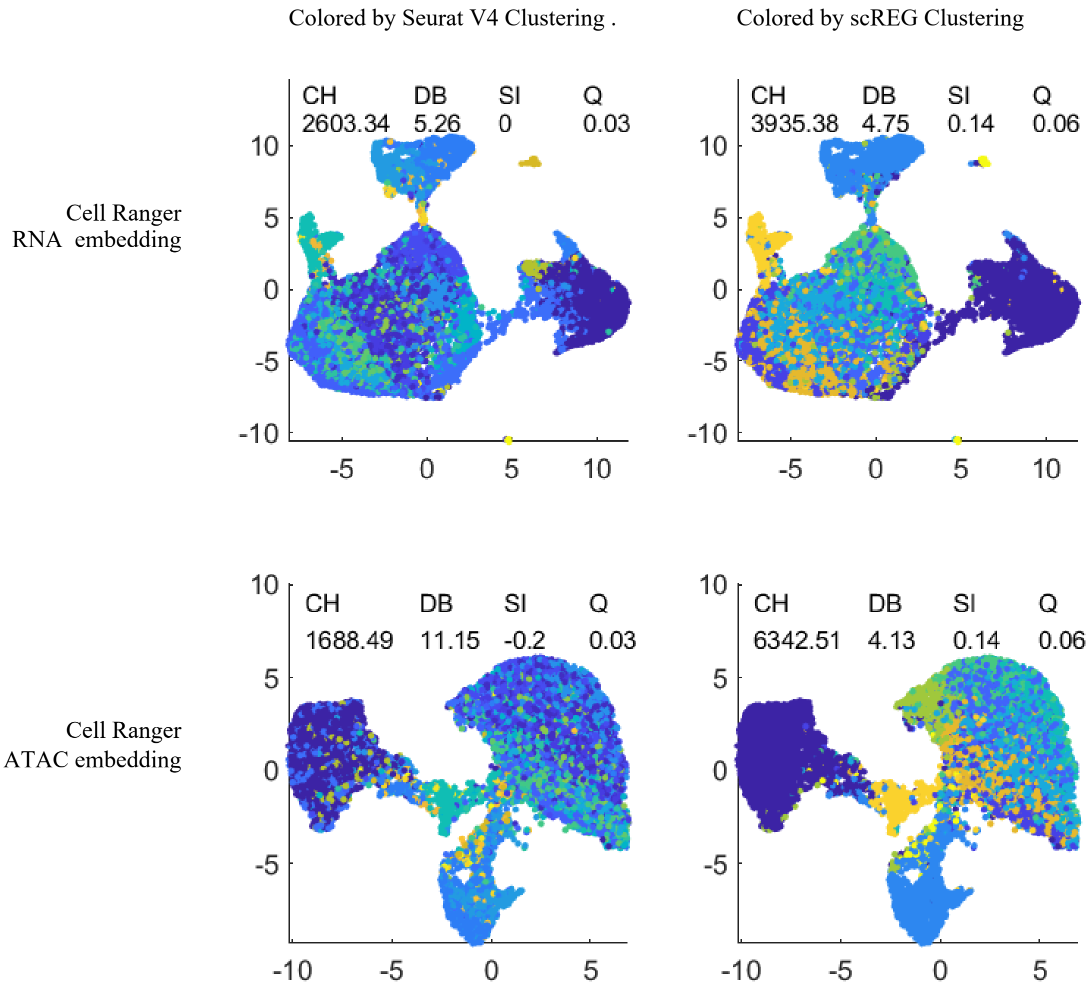

Figure S9. Evaluation of Seurat V4 clustering and scREG clustering on Cell Ranger scRNA-seq embedding and Cell Ranger scATAC-seq embedding on lymph node from B cell lymphoma. Each row presents one common UMAP embedding, and each column represents one clustering method. The color of the dot represent the clustering label. In each subfigures, consistency between different clustering label and 20-dimension PCs embedding are measured by calculating Calinski-Harabasz Index (CH), Davies-Bouldin Index (DB), Silhouette Index (SI), Modularity Q value (Q) respectively. A lower Davies-Bouldin index indicate better clustering, but the other three metrics are the higher the better.

S10

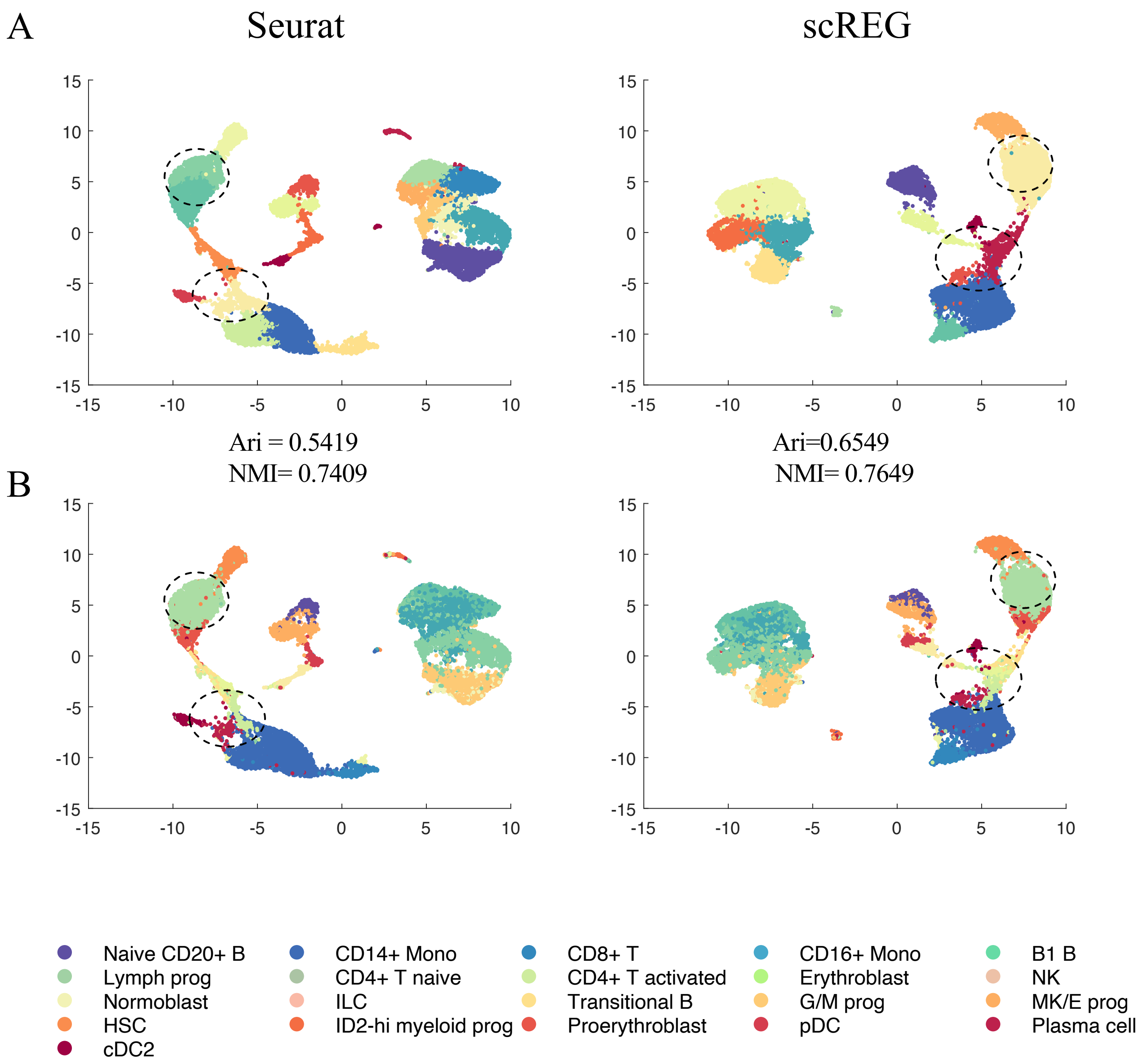

Figure S10. UMAP visualization of BMMC cell using Seurat and scREG clustering. (A) Scatter plot visualize the Umap embedding, colored by clustering label from different methods. (B) Same Umap as shown in (A) but colored by the surrogate ground truth. In Seurat clustering, we see the Lymph prog cell is separated into two clusters, while scREG is not separating it (upper circle). In Seurat+ clustering, Erythroblast cells are not separated from and Plasma cells (lower circle); while this separation is clear in scREG. Clustering performance also assessed by calculating Normalized Mutual Information (NMI) and Adjusted Rand Index (ARI) based on the surrogate ground truth.

S11

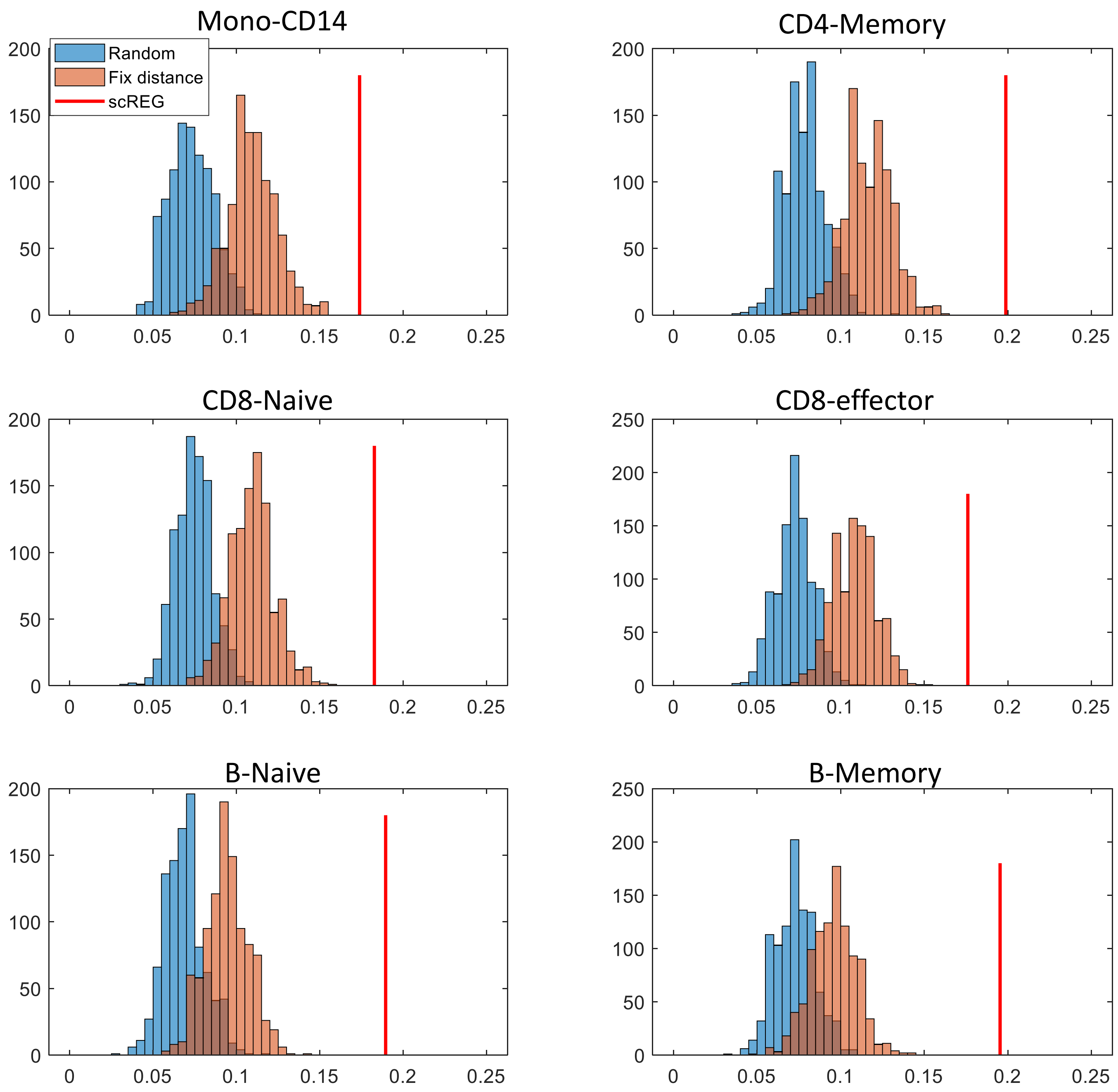

Figure S11. Validate the RE-TG prediction by HiC data. Consistency ratio of predicted RE and promoter capture HiC data on different cell types of. We can see in all cell type, scREG predict the greatest number of same RE-TG pairs as previously found promoter capture HiC data. set select distribution distance same with scREG, does improve the performance.

S12

		AUROC		AUPR		
Clusters	HiC Cell Type	scREG	PCC	scREG	PCC	Random
Mono-CD14	Mon	**0.637108**	0.544822	**0.164884**	0.129138	0.103509
CD4-Naive	nCD4	**0.629980**	0.535815	**0.178371**	0.117336	0.103074
CD4-Memory	aCD4	**0.631543**	0.535019	**0.153661**	0.119142	0.103261
CD8-Naive	nCD8	**0.644224**	0.563524	**0.166959**	0.137222	0.103023
CD8-effector	tCD8	**0.619746**	0.515763	**0.157308**	0.109361	0.102681
B-Naive	nB	**0.634953**	0.496358	**0.151975**	0.097066	0.090084
B-Memory	tB	**0.623901**	0.511744	**0.143048**	0.096570	0.094025

Figure S12. AUROC and AUPR predicting HiC data. Receiver operating characteristic (ROC) curve and Precision-recall (PR) curve taking the HiC data of 7 cell type as ground truth. Validate the prediction of scREG. Curves were plotted by sliding the predicted cis- regulatory score of peak-gene pair. Same as Figure 4(A), PCC was calculated as comparison to scREG prediction. We can see our method achieves much higher AUROC and AUPR than PCC on all cell type.

S13

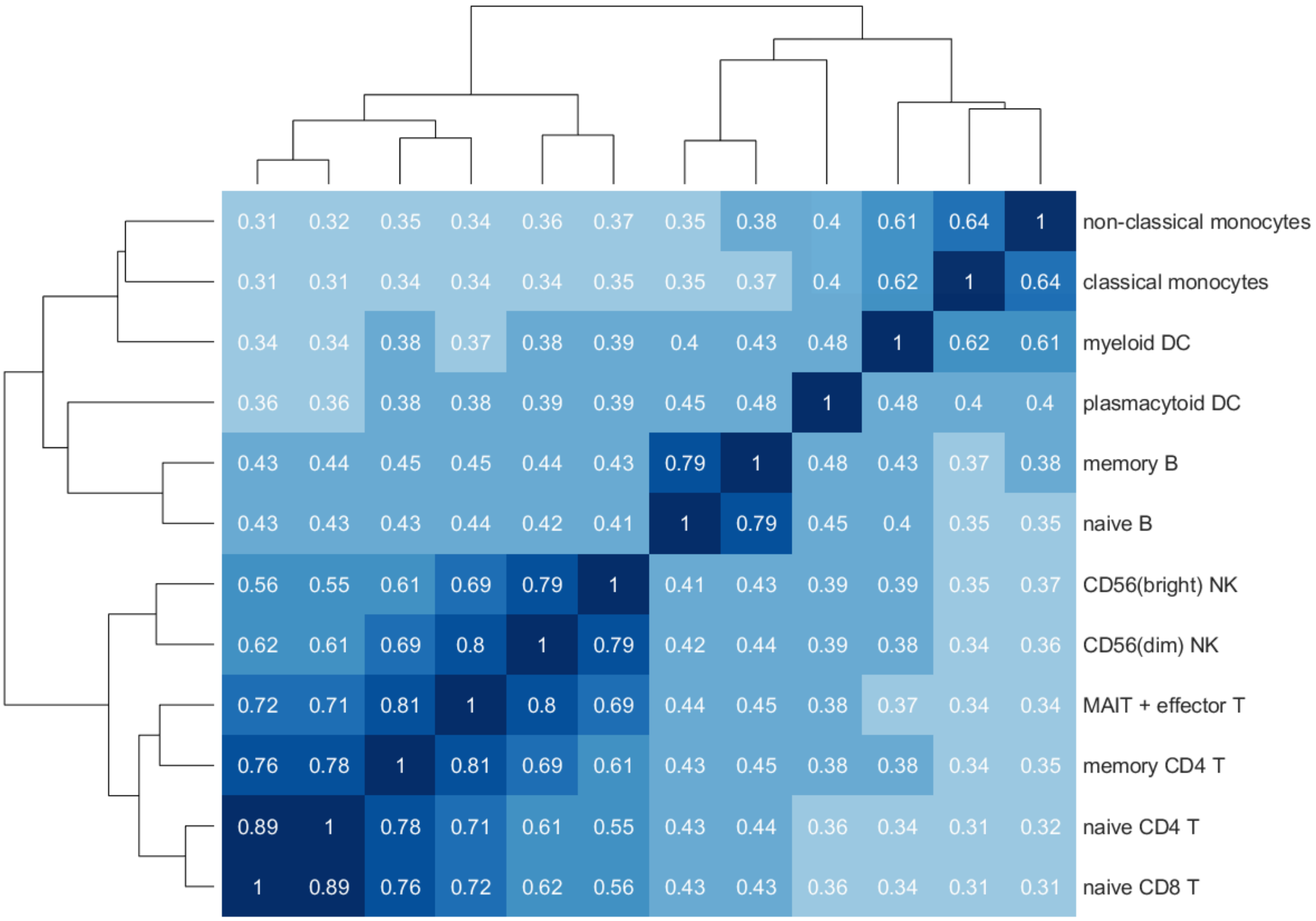

Figure S13. Clusters specificity of CRS. We select 10,000 RE-TG pairs with highest CRS score for each cluster and calculate Jaccard similarity between clusters. Some cis-regulation is conserved across cell type and some are cell type specific. The average Jaccard similarity between clusters are 0.4760.
