# Supplementary material for: Regulatory analysis of single cell multiome gene expression and chromatin accessibility data with scREG
Source: Genome Biol. 2022 May 16;23:114. doi: 10.1186/s13059-022-02682-2 (PMC9109353; doi:10.1186/s13059-022-02682-2)
Supplement: Supplementary file 2 — Additional file 2. Supplementary note for evaluation of embedding when the ground truth is not available. [file 13059_2022_2682_MOESM2_ESM.pdf]

Additional file2:

Supplementary Note: Regulatory analysis of single cell multiome gene expression and chromatin accessibility data with scREG

Duren et. al 2022

Evaluation of embedding when the ground truth is not available

The internal clustering evaluation metrics (i.e. Silhouette Index and Davies–Bouldin index) have been widely used for evaluating the clustering accuracy. However, such metrics cannot be used for evaluation of different embeddings (say produced by different dimension reduction methods). Because these metrics **are designed for evaluating different clustering results on a same embedding or on the same distance matrix.** When the distance matrices are different (from different methods), this type of comparison becomes meaningless. To illustrate this, we generate the following artificial examples.

Example 1: Silhouette Index only indicate consistency of clustering and the distance matrix that used to generate the clustering.

We simulated a distance among five cells B1, B2, B3 and T1, T2 (Figure R1 left). Here B1, B2, and B3 are three B cell, and the other two are two T cells. Based on this cell-cell distance matrix, we conduct Louvain clustering and obtain clustering label "1,1,1,2,2". This distance matrix provides a perfect clustering, but the average Silhouette index is only 0.7410.

If we adjust the distance matrics a little bit by shrinking the distance between T1-B3, and T2-B3 as 0.5 (Figure R2 right, the original distances are 2). Then, we conduct the Louvain clustering and obtain clustering label "1,1,2,2,2". The clustering has misclassified the B3 into the T cell cluster. However, the average Silhouette index is increased to 0.7533.

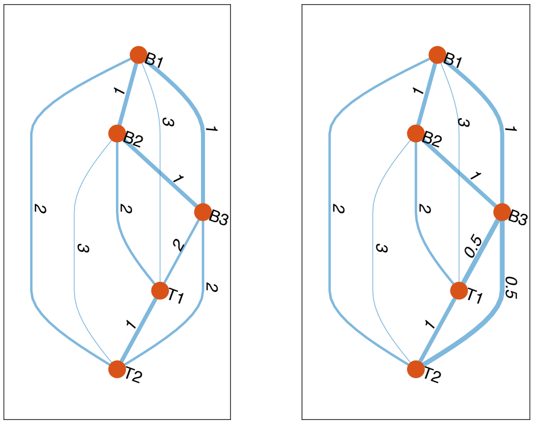

Figure R1. Plot shows the cell-cell distance of 5cells before and after adjusting.

From this example we can see Silhouette Index only indicate consistency of the clustering label and the distance matrix that used for generating the clustering label. In this case, an increased Silhouette index cannot reflect a more accurate clustering or a more accurate embedding.

Example 2 Internal clustering evaluation on single cell multiome data

We simulated a dataset consisting of 400 cells represented by 20 features (i.e. correspond to PCs in real data). There are 4 cell types and each has 100 cells. We generate four different 20-dimension embeddings and perform clustering analysis based on these different embeddings. Here is the characteristic of 4 embeddings (Please see the detail of data generation in the Appendix).

<u>Embedding 1:</u> Cell type 1 and 2 are separated clearly but cell type 3 and 4 are not separated. Simulating top 20 PCs of the scRNA-seq data.

<u>Embedding 2:</u> Cell type 1 and 2 are not separatable, but cell type 3 and 4 are separated clearly. Simulating top 20 PCs of the scATAC-seq data.

<u>Embedding 3:</u> All four cell types are separated. Simulating a good joint dimension reduction method.

<u>Embedding 4:</u> All four cell types could be detected but have bigger noise. Simulating a bad joint dimension reduction method.

In this example, some cell types are not separated in RNA-seq space, and some are not separated in ATAC-seq space. The following Figure shows the tSNE on the 4 embeddings colored by the true label. Since we have true label, we can first take a glimpse at the performance of the embeddings by calculating NMI and ARI.

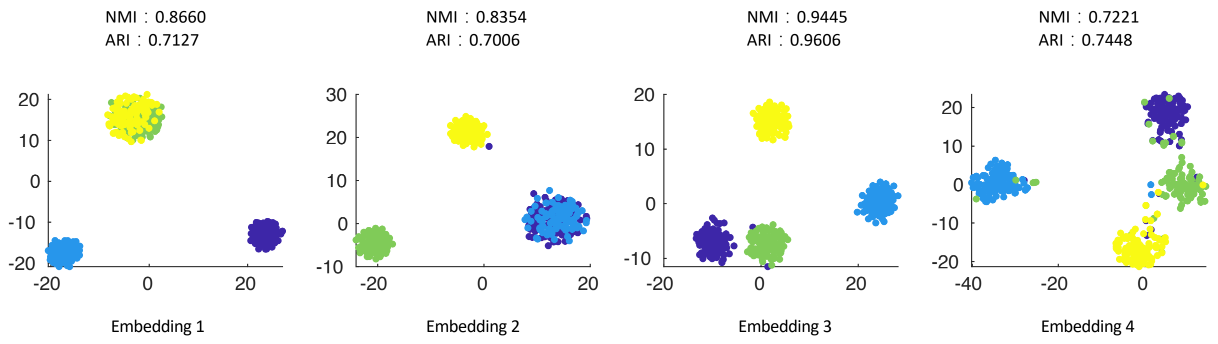

Figure R2. The tSNE plot of 4 different embeddings. Color represents the cell label.

From both the figure and the accuracy measurements, we can see the embedding 3 performs the best.

Now let's try to evaluate these embeddings imagining that we don't know the ground truth. If we do a Louvain clustering on these embeddings and calculate the internal clustering evaluation metrics (Calinski-Harabasz index, Davies-Bouldin index, silhouette index, and Q_modularity) based on the embeddings and their own clustering results, we got results like below.

	CH	DB	SI	Q
Embedding1	**483.8709**	**0.7930**	**0.7428**	**0.1639**
Embedding2	204.8519	1.2749	0.5127	0.1421
Embedding3	121.0004	1.5276	0.4163	0.0973
Embedding4	351.9862	0.9426	0.6429	0.1562

Based on the internal evaluation metrics, embeddings 3 performs the worst (DB index is the lower the better and the other 3 indexes are the higher the better). The results are not consistent with the evaluation based on the ground truth.

From this example, we can see the internal clustering evaluation metrics are not proper for comparing the clustering results based on different embedding data. Comparing clustering by internal clustering evaluation metrics should be done on the same embedding space (same distance matrix).

Now it is clear that clustering cannot be evaluated on their own embedding space, then the next question becomes which embedding space can provide a fair comparison between different embeddings. Here we propose to use RNA-seq alone embedding and ATAC-seq alone embedding to evaluate the clustering. If one clustering (say clustering based on embedding 3) is better than the other one (say clustering based on embedding 4) on **both** RNA-seq embedding (say embedding 1) based evaluation and ATAC-seq embedding (say embedding 2) based evaluation, we can say that clustering based on embedding 3 is better than clustering based on embedding 4.

Here we again use the toy example we created above to show this. Here, embedding 1 and 2 can be seen as two different modalities that capture different aspects of the variance of the data (like scRNA-seq and scATAC-seq), embedding 3 and 4 can be seen as two joint dimension reduction methods that capture both modality's information. We compare clustering 3 and 4 (which is based on embedding 3 and 4 respectively), by calculating internal clustering evaluation metrics with embedding 1 and 2. The following table shows that the clustering 3 performs better than clustering 4 on all 4 evaluation metrics under both embeddings. This example shows that our original validation is meaningful in this case.

CK	Clustering 3	Clustering 4
Embedding1	**312.3238**	208.7874
Embedding2	**124.6341**	69.1626

SI	Clustering 3	Clustering 4
Embedding1	**0.3912**	0.2876
Embedding2	**0.2646**	0.1469

DB	Clustering 3	Clustering 4
Embedding1	**3.1818**	3.7658
Embedding2	**4.4089**	5.5047

Q	Clustering 3	Clustering 4
Embedding1	**0.1136**	0.1093
Embedding2	**0.0964**	0.0881

Appendix: Data generation of the artificial example:

A. Cell labels

Cell 1-100 is cell type 1, cell 101-200 is cell type 2, cell 201-300 is cell type 3 and cell 301-400 is cell type 4.

B. Top 20 PCs (embeddings).

Embedding 1:

1) generate three cluster centers c1, c2, and c. They are 20-dimension vectors each independently generated from standard normal distribution.

2) The center of cell type 3 and cell type 4 are generated as c3=c+r3 and c4=c+r4, where r3 and r4 are generated from N(0.0.1).

3) embedding of cell type i is generated as ci+xi, where xi is 20*100 size of matrices generated from N(0,0.5).

Embedding 2:

1) generate three cluster centers c, c3, and c4. They are 20-dimension vectors each independently generated from standard normal distribution.

2) The center of cell type 1 and cell type 2 are generated as c1=c+r1 and c2=c+r2, where r1 and r2 are generated from N(0.0.1).

3) embedding of cell type i is generated as ci+xi, where xi is 20*100 size of matrices generated from N(0,0.8).

Embedding 3:

1) generate three cluster centers c1, c2, c3, and c4. They are 20-dimension vectors each independently generated from standard normal distribution.

2) embedding of cell type i is generated as ci+xi, where xi is 20*100 size of matrices generated from N(0,1).

Embedding 4:

1) generate three cluster centers c1, c2, c3, and c4. They are 20-dimension vectors each independently generated from standard normal distribution.

2) real embedding of cell type i is generated as ci+xi, where xi is 20*100 size of matrices generated from N(0,1.5).

3) KNN impute the real embedding: use nearest 20 cells' average real embedding to generate the new embedding. This will generate more structured embedding data.

C. Clusterings

We use Euclidean distance to calculate the distance between cells first and then calculate Jaccard distance based on their shared nearest neighbors (k=20). We cluster the cells using Louvain algorithm under the default settings.
