# Supplementary material for: Author Correction: Regulatory analysis of single cell multiome gene expression and chromatin accessibility data with scREG
Source: Genome Biol. 2022 Oct 13;23:213. doi: 10.1186/s13059-022-02786-9 (PMC9563437; doi:10.1186/s13059-022-02786-9)
Supplement: Supplementary file 1 — Additional file 1: Supplementary Figure S11. Validation of RE-TG prediction by HiC data. Consistency ratio of predicted RE and promoter capture HiC data on different cell types of. We can see in all cell type, scREG predict the greatest number of same RE-TG pairs as previously found promoter capture HiC data. set select distribution distance same with scREG, does improve the performance. Supplementary Figure S12. AUROC and AUPR of RE-TG predictio by taking HiC data as ground truth. [file 13059_2022_2786_MOESM1_ESM.docx]

Supplementary Figure S11. Validation of RE-TG prediction by HiC data. Consistency ratio of predicted RE and promoter capture HiC data on different cell types of. We can see in all cell type, scREG predict the greatest number of same RE-TG pairs as previously found promoter capture HiC data. set select distribution distance same with scREG, does improve the performance.

**Mono-CD14**

**CD4-Memory**

**CD8-effector**

**CD8-Naive**

**B-Memory**

**B-Naive**

Supplementary Figure S12. AUROC and AUPR of RE-TG predictio by taking HiC data as ground truth.

|  |  | AUROC | | AUPRC | | |
| --- | --- | --- | --- | --- | --- | --- |
| Clusters | HiC Cell Type | RegNMF | PCC | RegNMF | PCC | Random |
| Mono-CD14 | Mon | **0.712671** | 0.526481 | **0.389114** | 0.247010 | 0.230167 |
| CD4-Naive | nCD4 | **0.729035** | 0.554712 | **0.376779** | 0.239046 | 0.209318 |
| CD4-Memory | aCD4 | **0.726929** | 0.562834 | **0.367472** | 0.250804 | 0.213716 |
| CD8-Naive | nCD8 | **0.721787** | 0.563092 | **0.359063** | 0.240744 | 0.204817 |
| CD8-effector | tCD8 | **0.728576** | 0.561769 | **0.363729** | 0.246634 | 0.208963 |
| B-Naive | nB | **0.685907** | 0.527016 | **0.304047** | 0.216047 | 0.200303 |
| B-Memory | tB | **0.714880** | 0.538674 | **0.355491** | 0.230196 | 0.209479 |
